# Genome sequence of a novel phlebovirus associated with lettuce big vein disease infecting lettuce (*Lactuca* spp.)

**DOI:** 10.1128/mra.00979-23

**Published:** 2023-12-22

**Authors:** Willem E. W. Schravesande, Peter de Heer, Adriaan Verhage, Harrold A. van den Burg

**Affiliations:** 1 Molecular Plant Pathology, Swammerdam Institute for Life Sciences (SILS), University of Amsterdam, Amsterdam, the Netherlands; 2 Rijk Zwaan Breeding B.V., Burgemeester Crezéelaan, De Lier, the Netherlands; DOE Joint Genome Institute, Berkeley, California, USA

**Keywords:** virology, plant viruses, plant pathogens, plant-microbe interactions, genomes

## Abstract

Lettuce big vein disease is a disease complex involving at least two RNA viruses, both transmitted by the soilborne fungus *Olpidium virulentus*. Here, we present the genomic sequence of a novel unrelated third negative-stranded RNA virus, belonging to the family *Phenuiviridae*, recovered from infected lettuce plants.

## ANNOUNCEMENT

Lettuce big vein disease (LBVD) is a damaging soilborne viral disease that lowers yield and quality of lettuce production in cold seasons worldwide. Symptomology includes vein-band chlorosis, stunted growth, crinkled leaves, and absence of heads (1). LBVD is associated with two unrelated negative-stranded RNA viruses, lettuce big vein associated virus (LBVaV) and Mirafiori lettuce big vein virus (MiLBVV) (2–4).

Previous studies did not result in an unambiguous answer regarding the respective roles of MiLBVV and LBVaV (5–9). To gain a better understanding of the roles of the individual viruses, a collection of soil samples of LBVD-infected fields was subjected to metagenomic sequencing upon re-infection of lettuce plants. In this study, we report the genome sequence of a novel third negative-stranded RNA virus, a phlebovirus, retrieved from infected plant material associated with LBVD.

Soil samples originating from symptomatic plants were used to perform re-infections on *Lactuca sativa* cv. Iglo. In short, plants were sown on soilblocks and grown at 14°C for 2 weeks. The seedlings were transplanted into a hydroponic gutter system where water containing virus-containing *O. virulentus* spores is circulating. After 12 weeks, the plants were sampled. Plants that tested positive for the presence of either MiLBVV or LBVaV by reverse transcription-quantitative polymerase chain reaction (RT-qPCR) were selected for sequencing (Table 1). In total, 27 individual samples were selected for total RNA sequencing. Leaf material was grinded to a fine powder using mortar and pestle and used as input material for total RNA isolation using the RNeasy Plant Mini Kit (Qiagen). The isolated total RNA was used for cDNA synthesis using the Maxima H Minus Double Stranded cDNA Synthesis Kit using random hexamer primers (Thermo Scientific). For library construction, the Ligation Sequencing Kit SQK-LSK110 was used in combination with the Native Barcoding Kits EXP-NBD104 and EXP-NBD114 (Oxford Nanopore) on the synthesized cDNA without further size selection and/or shearing. Manufacturer’s instructions and protocols were followed. The constructed sequencing libraries were sequenced using the MinION FLO-MIN106D flowcells (Oxford Nanopore) during a period of 72 hours (N_50_, 572; total reads, 19,224,566).

**TABLE 1:** Primer sequences used for RACE PCR and RT-qPCR

Primer name	Primer sequence 5′–3′
Phe_R1_SP1	ACACCTCTAGGATTGACTATGA
Phe_R1_SP2	TTAGATGAAACGGCTAGTATGG
Phe_R1_SP5	CCGACACCAAGAATCAAGTAT
Phe_R2_SP1	TTTGGTCGACTGAGCTTAAC
Phe_R2_SP2	GCTGGATTTGTCCTTGAGATA
Phe_R2_SP5	CAAAGACCACCTTCTCATCTT
Phe_R3_SP1	GTAGGCCAAGGAATACTGAAA
Phe_R3_SP2	GAACTGATCAACACTCTCATACA
Phe_R3_SP5	CTTGGCTGAGGAGAAAGTTAG
Phe_R4_SP1	TGAAGCCAGAAGGGTAATAAAG
Phe_R4_SP2	GTGCCTTCCAAGAGGATTC
Phe_R4_SP5	GCCCTACCGACTCTATCAA
MiLBVV-167F	AATTTCTYTWGGTCTCATGACAA
MiLBVV-238R	TTTGCAGATGCYACCATGG
MiLBVV-205T	FAM-ACAGGCTTCTCTTC-MGB-NFQ
LBVaV-F	TGGTAGAGACCAATGTGAAGGA
LBVaV-R	CGGTCTTACAGAAATCAGCATACC
LBVaV-P	HEX-AGAGCCGGAGCATTCATCGCTGC-BHQ1

The raw sequencing data were basecalled (Guppy v.3.6, high-accuracy mode) and subjected to QC filtering [Nanofilt v.2.2.0 (10)], barcode demultiplexing, and adapter trimming (qcat v1.1.0). Reads originating from the host were subtracted by mapping to an in-house-generated reference genome of *L. sativae* cv. Iglo [minimap2 v.2.16 (11)]. The unmapped reads were used for *de novo* genome assembly [Canu v1.9 (12)]. The genome assemblies were polished using Medaka v.1.0.3, followed by manual curation and annotation. The true 5′/3′ termini of the RNA segments were obtained using rapid amplification of cDNA ends (5′/3′RACE Kit, 2nd Generation, Roche) (Table 1).

**Fig 1:**
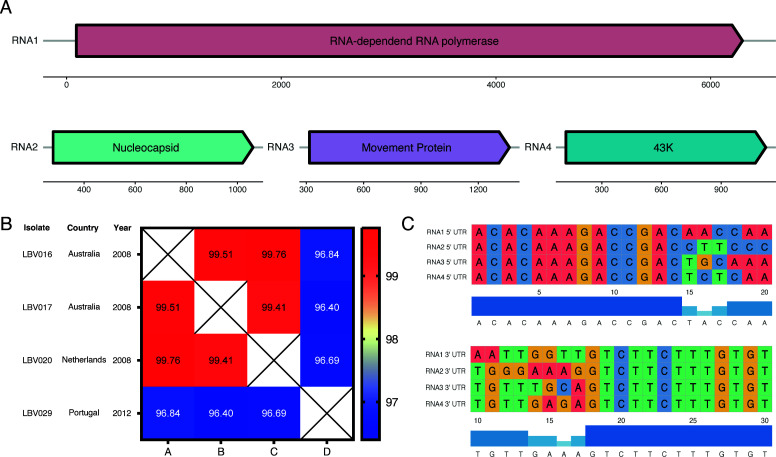
(**A**) Schematic overview of novel phlebovirus. RNA segments are depicted as viral complement RNA with the putative ORFs. (**B**) Overview of isolates of the novel phlebovirus identified in the sequenced LBVD collection. Heatmap visualizes the percentage identity between the different specimens (Geneious Prime v.2022.2.2). (**C**) Alignment of 5′- and 3′-UTRs of the four RNA segments, containing the conserved motif found in phleboviruses.

The assembled contigs were compared to a custom database containing all RefSeq genomes of plant infecting viruses using BLAST (13–15), leading to the classification of the contigs to MiLBVV, LBVaV, and a novel virus associated with the *Phenuiviridae* family. The assembled genome consisted of four RNA segments of 6,408 (40.6× coverage), 1,107 (134.7×), 1,429 (99.5×), and 1,282 (24.4×) nucleotides and a GC content of 35.9% ( Fig. 1). The virus has four predicted open reading frames (ORFs) that putatively code for the RNA-dependent RNA polymerase, nucleocapsid, putative movement protein, and a 43K protein of unknown function (Geneious Prime v.2022.2.2). This annotation is based on sequence similarity with another phlebovirus, Tulip streak virus (LC571987.1, LC571988.1), and protein structure comparisons to the PDB database using Foldseek (16). This novel virus was found in four independent samples of the sequenced isolate collection. Further research is needed to fully comprehend the potential role(s) of this novel virus in both infection transmission and symptomology. We propose the name “Lactuca Big Vein associated Phlebovirus (LBVaPV)” for this novel phlebovirus.

## Data Availability

The information and genomic sequence have been deposited in GenBank under the accession number OR610326/OR610327/OR610328/OR610329. The raw reads were deposited under BioProject accession number PRJNA1043872.
